# First use of ^18^F-FDG PET in TEMPI syndrome: can it be used for treatment assessment? A case report

**DOI:** 10.3389/fnume.2023.1273967

**Published:** 2023-10-26

**Authors:** Henri Pasquesoone, Aurélien Callaud, Thibaut Carsuzaa, Thomas Chalopin, Maria-Joao Santiago-Ribeiro

**Affiliations:** ^1^ Nuclear Medicine Department, CHRU TOURS, Tours, France; ^2^ Hematology Department, CHRU Tours, Tours, France; ^3^ UMR 1253, iBrain, Université de Tours, Inserm, Tours, France

**Keywords:** TEMPI, ^18^F-FDG, PET, multifocal hypermetabolic bone lesions, case report

## Abstract

TEMPI syndrome (TEMPI) compounds telangiectasias and polycythemia with elevated erythropoietin levels, monoclonal gammopathy, perirenal fluid collections, and intrapulmonary shunt. Although the pathophysiology of this syndrome remains unclarified, prior research has been established that it is a plasma cell neoplasm, often containing less than 10% bone marrow plasma cells. ^18^F-FDG PET serves as a valuable instrument for initial staging and treatment monitoring in multiple myeloma management. Thus, ^18^F-FDG PET can be legitimately applied for TEMPI assessment. Here, we present the first ^18^F-FDG PET images for the initial evaluation and treatment monitoring of TEMPI in a 51-year-old woman, who exhibited polycythemia (EPO:5,448 mIU/ml) without JAK2 mutation, telangiectasias, monoclonal IgG lambda gammopathy (13.9) g/L and 7% dysmorphic plasma cells (CD38 + CD138+), occasionally clustered, in favor of tumoral plasmacytomas. The first PET scan exhibited hypermetabolic diffuse bone marrow, potentially related to polycythemia, accompanied by non-lytic bone hypermetabolic lesions in the femoral and humeral diaphysis, and ametabolic peri-renal fluid collections, brown fat, and pleural talcoma. Post-treatment ^18^F-FDG PET (Daratumumab Bortezomib Thalidomide Dexamethasone) revealed a completely reduced signal of bone lesions, suggesting a complete response, which was substantiated both clinically and biologically, with the concurrent disappearance of telangiectasia and the monoclonal component, and the normalization of the EPO level. In future, additional data will be required to confirm the added value of ^18^F-FDG PET with TEMPI. Nevertheless, ^18^F-FDG PET can be a preferred tool for the extension workup and therapeutic evaluation of TEMPI syndrome.

## Introduction

We’re discussing here a TEMPI syndrome case, which is a condition characterised by a syndrome compound by telangiectasias and polycythemia with elevated erythropoietin levels, monoclonal gammopathy, perirenal fluid collections, and intrapulmonary shunt (1–4). Although the pathophysiology of this syndrome remains unclarified, prior research has been established that it is a plasma cell neoplasm, often containing less than 10% bone marrow plasma cells. ^18^F-FDG PET serves as a valuable instrument for initial staging and treatment monitoring in multiple myeloma management. Thus, ^18^F-FDG PET can be legitimately applied for TEMPI assessment.

## Case report (including patient information and clinical findings, diagnostic, therapeutic follow-up, and outcomes)

**Figure 1:**
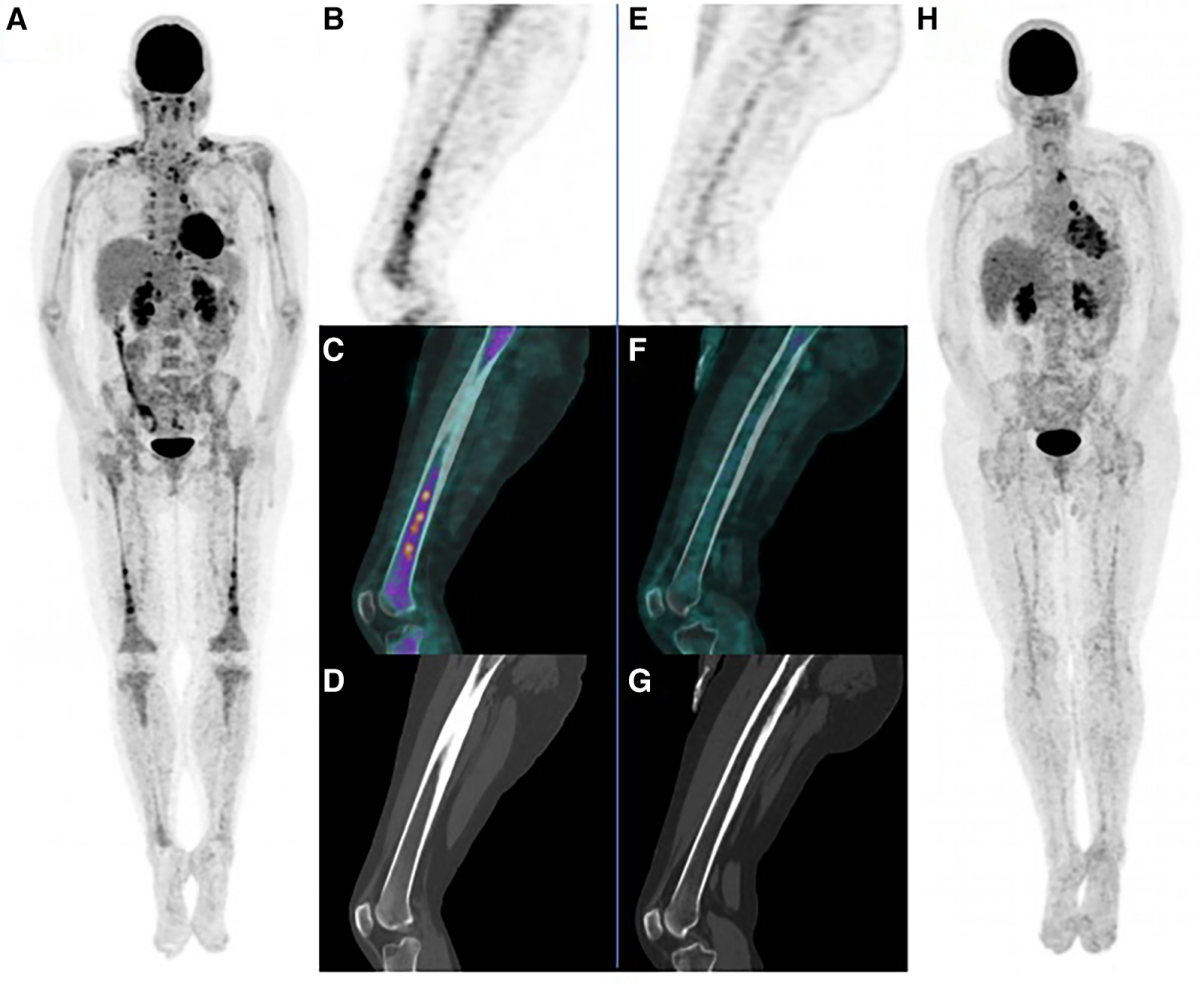
First PET scan with maximal intensity projection (MIP) (**A**), sagittal PET (**B**), fusion (**C**), and CT (**D**). Final post treatment PET scan with MIP (**H**), sagittal PET (**E**), fusion (**F**), and CT (**G**), showing completely reduced signal of bone lesions, and almost complete peri-renal collection response.

Here, we present the first ^18^F-FDG PET images for the initial evaluation and treatment monitoring of TEMPI in a 51-year-old woman, with history of splenic artery thrombosis in 2017, leading to the discovery of JAK2-negative polycythemia, with monoclonal IgG lambda gammopathy (13.9 g/L), low bone marrow plasmacytosis (5%), and telangiectasias leading to the diagnosis of TEMPI syndrome with 3 major criteria and one minor criteria (2). No exploration for shunt were performed at this time, and pluri-disciplinary decision was made for surveillance with anticoagulant therapy only. In October 2021, the patient presented fever spikes and suspected pneumopathy leading to hospitalization, with persistent telangiectasias and a preserved general condition. She exhibited polycythemia (EPO:5,448 mUI/ml) and a plasmacytosis at 7% with 0.23% of dysmorphic plasma cells (CD38 + CD138 +), occasionally clustered, in favor of tumoral plasma cells (5). A first ^18^F-FDG PET scan was performed for infectious etiological assessment, and it exhibited hypermetabolic diffuse bone marrow, potentially related to polycythemia, accompanied by non-lytic bone hypermetabolic lesions in the femoral and humeral diaphysis, ametabolic peri-renal fluid collections, brown fat, and pleural talcoma (Figure 1: maximal intensity projection (MIP) (A), sagittal PET (B), fusion (C), and CT (D)). These lesions were similar to lesions associated to bone marrow hyperplasia (6), lymphomas, myelomas, POEMS syndrome (7) or other hematologic malignancy (8), but through the overall clinical presentation, its character direcly related to TEMPI was retained. Thus, considering anemia and hypermetabolic foci, pluridisciplinary decision was made to initiate a treatment with 6 cycles of Daratumumab (1,800 mg), Bortezomib (2.25 mg), Thalidomide (100 mg) and Dexamethasone (40 mg) (9–11) (D-VTD treatment). It was started in November 2021, for 6 cycle each 3 weeks, and stopped in August 2022, without any severe side effect, adverse or unanticipated events.

In September 2022, a post-treatment ^18^F-FDG PET was performed to evaluate metabolic lesions response to treatment. showed a completely reduced signal of bone lesions, suggesting a complete response (Figure 1: MIP (H), sagittal PET (E), fusion (F), and CT (G)), which was substantiated both clinically and biologically, with the concurrent disappearance of telangiectasia and the monoclonal component, and the normalization of the EPO level (12). Peri-renal collection had an almost complete response with only a thin strip of left perirenal non hypermetabolic effusion remaining.

## Discussion and patient perspective

**Figure 2:**
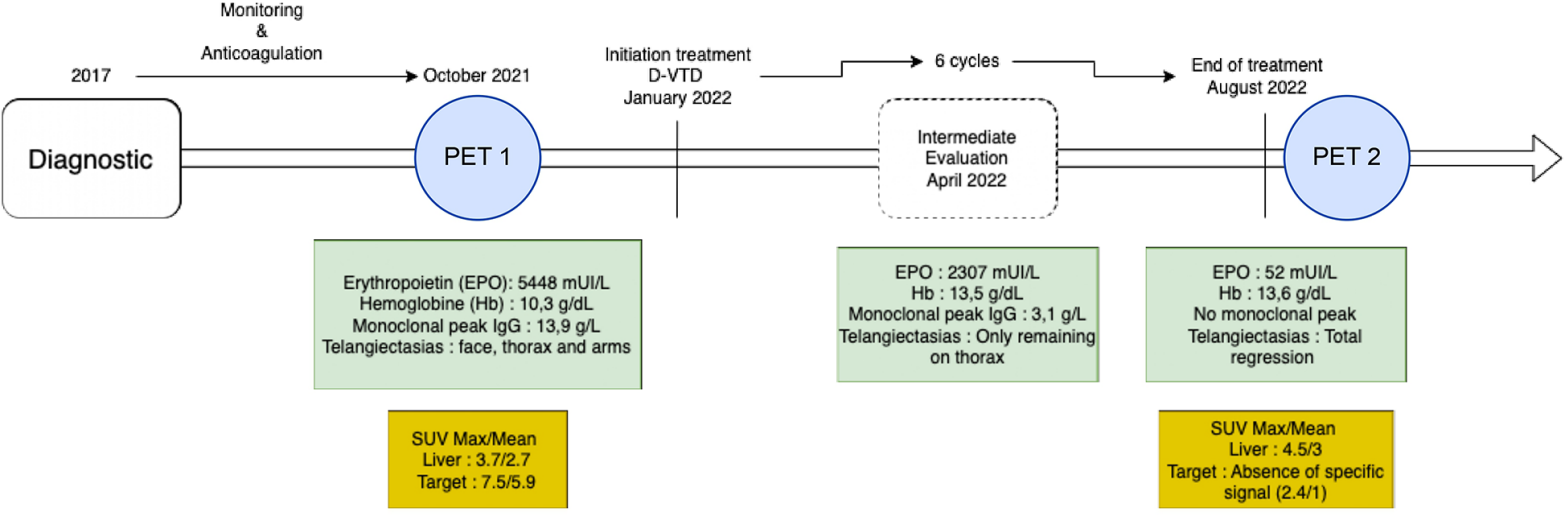
Timeline.

Chronology of markers and SUVmax evolution during treatment is summarized in Figure 2. There is a need for additional data, that will be required to confirm the potential added value of ^18^F-FDG PET with TEMPI. Nevertheless, ^18^F-FDG PET can be a preferred tool for the extension workup and therapeutic evaluation of TEMPI syndrome. The patient was reassured by the hypermetabolic lesion's disappearance on ^18^F-FDG PET scan, with a reported added value for her, compared with only clinical and biological assessment.

## Data Availability

The raw data supporting the conclusions of this article will be made available by the authors, without undue reservation.

## References

[1] Xu Z-F Ruan J Chang L Wu S Lin J Wang W Case report: TEMPI syndrome: report of three cases and treatment follow-up. Front Oncol. (2022) 12:949647. 10.3389/fonc.2022.949647 35992844 PMC9382296

[2] Sykes DB Schroyens W O’Connell C. The TEMPI syndrome–a novel multisystem disease. N Engl J Med. (2011) 365:475–7. 10.1056/NEJMc1106670 21812700

[3] Sykes DB O’Connell C Schroyens W. The TEMPI syndrome. Blood. (2020) 135:1199–203. 10.1182/blood.2019004216 32108223

[4] Xu J Liu W Fan F Zhang B Zhao F Hu Y TEMPI syndrome: update on clinical features, management, and pathogenesis. Front Endocrinol (Lausanne). (2022) 13:886961. 10.3389/fendo.2022.886961 35663307 PMC9161818

[5] Zhang X Fang M. TEMPI syndrome: erythrocytosis in plasma cell dyscrasia. Clin Lymphoma Myeloma Leuk. (2018) 18:724–30. 10.1016/j.clml.2018.07.284 30100329

[6] Kwee TC De Klerk JMH Nix M Heggelman BGF Dubois SV Adams HJA. Benign bone conditions that may be FDG-avid and mimic malignancy. Semin Nucl Med. (2017) 47:322–51. 10.1053/j.semnuclmed.2017.02.004 28583274

[7] Pan Q Li J Li F Zhou D Zhu Z. Characterizing POEMS syndrome with ^18^F-FDG PET/CT. J Nucl Med. (2015) 56:1334–7. 10.2967/jnumed.115.160507 26182964

[8] Adams HJA De Klerk JMH Heggelman BGF Dubois SV Kwee TC. Malignancy rate of biopsied suspicious bone lesions identified on FDG PET/CT. Eur J Nucl Med Mol Imaging. (2016) 43:1231–8. 10.1007/s00259-015-3282-4 26728144 PMC4865543

[9] Kwok M Korde N Landgren O. Bortezomib to treat the TEMPI syndrome. N Engl J Med. (2012) 366:1843–5. 10.1056/NEJMc1202649 22571216 PMC6824588

[10] Kawamura S Tamaki M Nakamura Y Kawamura M Takeshita J Yoshino N Successful treatment of the TEMPI syndrome with pomalidomide plus dexamethasone followed by autologous stem cell transplantation. Acta Haematol. (2022) 145:553–9. 10.1159/000525056 35605591

[11] Undar L Atas U Iltar U Salim O Yucel OK Alpsoy E. Long-term complete clinical and hematological response with bortezomib: the report of a case with TEM(P)I syndrome and a review of the literature. Clin Lymphoma Myeloma Leuk. (2022) 22:702–7. 10.1016/j.clml.2022.04.018 35624059

[12] Rosado FG Oliveira JL Sohani AR Schroyens W Sykes DB Kenderian SS Bone marrow findings of the newly described TEMPI syndrome: when erythrocytosis and plasma cell dyscrasia coexist. Mod Pathol. (2015) 28:367–72. 10.1038/modpathol.2014.117 25216227

